# Metabolic Targeting of Cancer Stem Cells

**DOI:** 10.3389/fonc.2020.537930

**Published:** 2020-12-22

**Authors:** Anna Mukha, Anna Dubrovska

**Affiliations:** ^1^ OncoRay-National Center for Radiation Research in Oncology, Faculty of Medicine and University Hospital Carl Gustav Carus, Technische Universität Dresden and Helmholtz-Zentrum Dresden-Rossendorf, Dresden, Germany; ^2^ Helmholtz-Zentrum Dresden - Rossendorf, Institute of Radiooncology – OncoRay, Dresden, Germany; ^3^ German Cancer Consortium (DKTK), Partner Site Dresden, Dresden, Germany; ^4^ German Cancer Research Center (DKFZ), Heidelberg, Germany

**Keywords:** cancer stem cells, therapy resistance, metabolic targeting, OXPHOS, glycolysis, glutamine metabolism, fatty acid metabolism, tumor microenvironment

## Abstract

Most human tumors possess a high heterogeneity resulting from both clonal evolution and cell differentiation program. The process of cell differentiation is initiated from a population of cancer stem cells (CSCs), which are enriched in tumor‐regenerating and tumor‐propagating activities and responsible for tumor maintenance and regrowth after treatment. Intrinsic resistance to conventional therapies, as well as a high degree of phenotypic plasticity, makes CSCs hard-to-target tumor cell population. Reprogramming of CSC metabolic pathways plays an essential role in tumor progression and metastatic spread. Many of these pathways confer cell adaptation to the microenvironmental stresses, including a shortage of nutrients and anti-cancer therapies. A better understanding of CSC metabolic dependences as well as metabolic communication between CSCs and the tumor microenvironment are of utmost importance for efficient cancer treatment. In this mini-review, we discuss the general characteristics of CSC metabolism and potential metabolic targeting of CSC populations as a potent strategy to enhance the efficacy of conventional treatment approaches.

## Introduction

According to the world health organization (WHO), cancer is responsible for one in six deaths worldwide, and global cancer rates continue to grow (1, 2). Although the mono-therapy such as surgery, chemotherapy and radiotherapy is a commonly accepted treatment modality for different types of cancers, the combination of two or more types of treatment targeting the key cancer mechanisms in synergistic or additive manners is currently a cornerstone of anticancer therapy especially for advanced and aggressive cancers (3, 4). Recent innovations in treatment technologies as well as in precision of radiation and drug delivery substantially increased efficiency and quality of treatment. However, treatment-related toxicities and tumor therapy resistance still constitute a fundamental clinical and scientific challenge (5–7).

The difficulty of cancer treatment has its roots in the nature of this disease. Tumors are highly heterogeneous, consisting of different types of cells. Intratumoral heterogeneity is evidenced at the multiple levels, including genetic and epigenetic landscapes, histological and molecular specificities as well as functional differences between tumor cells including their abilities to propagate tumor growth and give rise to other types of cancer cells by the process of differentiation (8).

The process of cell differentiation is initiated from a population of cancer stem cells (CSCs) that possess unique properties such as the unlimited capacity of self-renewal and asymmetric division, which leads to the production of different cell types within tumors. These properties of CSCs make them equipped with tumor‐regenerating and tumor‐propagating activities and, therefore, responsible for the tumor maintenance and regrowth after treatment. The density of CSCs substantially varies between individual tumors, and its analysis is proven to have prognostic significance for different types of cancers (9, 10). Several CSC-specific markers have been described, among them the expression of CD133, CD44, CD117 (c-kit), Oct4, high aldehyde dehydrogenase (ALDH) activity, etc. as discussed elsewhere (11–13). However, some of these markers can be found in normal stem cells, which make identification and targeting of CSCs more challenging (14). A high plasticity of CSC populations is an additional obstacle on the way of clinical translation as tumor cells possess the ability of shifting their state from the CSC- to non-CSC populations and vice versa that is regulated by multiple genetic, epigenetic and microenvironmental stimuli (15–18). Although tumor stemness is described as a highly dynamic state, eradication of all CSC populations during tumor treatment is of high clinical importance as remaining CSCs might re-initiate local tumor growth and lead to metastatic dissemination.

Many preclinical and clinical studies suggested that some CSC populations can be equipped with intrinsic and extrinsic mechanisms providing them with high radioresistance and chemoresistance compared to the bulk of tumor cells. This relatively high therapy resistance of CSCs is attributed to the efficient DNA repair, low proliferative rate, protective tumor microenvironment, maintenance of cellular redox homeostasis, and immune escape. Altered metabolism of CSCs substantially contributes to their treatment resistance. A deep understanding of the CSC metabolic features and their molecular background will help to develop novel therapeutic strategies that precisely target CSCs and improve the efficiency of cancer control.

## Metabolic Characteristics of CSCs

Reprogramming of cellular metabolism plays a crucial role in tumor initiation, progression, resistance to conventional therapy, and immunosuppression. Unique features of tumor metabolism were noticed almost one hundred years ago. At the beginning of the XX century, Otto Warburg and co-workers described aerobic glycolysis, accompanied by excessive production of lactate, as one of the distinct characteristics of tumor cells and tissue slices (19). Since then, many other alterations of biochemical pathways have been described for cancer cells (11, 20, 21). Studying the metabolism of CSCs is a challenging task due to the small size and high plasticity of these cell populations. Nevertheless, current experimental data shows that the metabolic features of CSCs are highly heterogeneous, and tumor type-dependent (**Table 1**).

**Table 1:** Examples of the metabolic features of CSCs described for the different tumor models.

Metabolic feature of CSCs	Tumor entity	Model	Potential therapeutic targets	References
Glycolysis	Hepatocellular carcinoma	PLC/PRF/5 human hepatocellular cancer cell line; CD133+ subpopulation was obtained by cell sorting	n/a	(22)
	Osteosarcoma	OS13 cell line established by authors; CSC population was obtained by limiting dilution assay *in vitro*	LIN28	(23)
	Breast cancer	Tumor-initiating cells purified from MMTV-*Wnt-1* murine breast tumors	Decreased activity of pyruvate dehydrogenase (Pdh)	(24)
	Breast cancer	CD44^+^/CD24^−^ breast cancer stem cells	Pyruvate dehydrogenase kinase (PDK1)	(25)
Glycolysis and OXPHOS	Lung cancer	CSC-like cells enriched under sphere forming conditions	Glycolysis itself (inhibition with 2-deoxyglucose reduced CSC features)	(26)
	Esophageal cancer	CSC-like cells enriched under sphere forming conditions	HSP27, HK2	(27)
OXPHOS	Glioblastoma	CD133+ CSCs from glioma spheres	IMP2	(28)
	Acute myeloid leukemia	Primary AML patient-derived cells; ROS-low CSC population was isolated by cell sorting	BCL-2	(29)
	Lung cancer	CSCs derived from A549 lung cancer cell line by using single-cell cloning culture	n/a	(30)
	Pancreatic cancer	CD133+ cells derived from patient samples	Mitochondrial complex I (targeted with metformin)	(31)
	Ovarian cancer	CD44+ CD117+ cells from ascitic fluid of ovarian cancer patients	Mitochondrial complex I	(32)
	Breast cancer	MCF7 and MDA-MB-231 cells; CSC-like cells enriched under sphere forming conditions	Mitochondrial respiration	(33)
De novo fatty acid synthesis	Glioma	Patient-derived glioblastoma cell lines; CSC population was enriched by culturing cell lines in serum-free neurobasal medium	FASN (fatty acid synthase)	(34)
	Breast cancer	Epithelial CSCs derived from MCF10A cells; patients’ tissue samples; CD24- CD44+ ESA+ CSC-like cells were isolated by magnetic-activated cell sorting	SREBP1 (targeted with resveratrol)	(35)
	Breast cancer	ERBB2-positive breast cancer cells; CSC-like cells were sorted as side population (SP); CSC signature of ERBB2-positive cells was confirmed by high ALDH activity	PPARγ pathway	(36)
	Pancreatic cancer	CSCs derived from Panc1 cell line and enriched under sphere-forming conditions	FASN (targeted with cerulenin); mevalonate pathway (targeted with atorvastatin)	(37)
Glutamine metabolism	Pancreatic cancer	PDAC cells	CD9	(38)
	Non-small cell lung cancer Pancreatic cancer Glioblastoma	Side population of cell lines: A549 AsPC-1 GSC11 GSC23	n/a	(39)
	Neuroblastoma	Cell lines BE(2)-C, SH-SY5Y and SK-N-AS	MycN and c-Myc	(40)
	Hepatocellular carcinoma	Publicly available data from Cancer Genome Atlas; Cell lines HCCLM3 and HC22; Tumor tissue samples from HCC patients	GLS1	(41)

Glycolysis is one of the major and best-studied metabolic characteristics of cancer cells. Fast-growing tissues, such as the most malignant tumors, demand more energy. In differentiated cells, energy in the form of adenosine triphosphate (ATP) is produced *via* oxidative phosphorylation (OXPHOS) that occurs in mitochondria. Complete oxidation of glucose molecule leads to the production of about 30 molecules of ATP, whereas about 26 out of these 30 ATP molecules are generated by OXPHOS (42). Fast-proliferating cancer cells switch from OXPHOS to glycolysis that requires the consumption of a high amount of glucose since only two molecules of ATP per one molecule of consumed glucose can be produced *via* this pathway. Lactate, a byproduct of aerobic glycolysis, is shuttled to the extracellular space and was shown to support stemness by upregulation of the expression of genes related to stem cell properties, such as transcription factor SP1, sterol regulatory element-binding protein 1 (SREBP1) which is a transcriptional activator required for regulation of lipid homeostasis, etc., to increase aggressiveness and invasive properties of cancer cells as well as to promote immunosuppression (43–48). Glycolytic CSCs were described for several tumor entities. Song et al. showed that CD133+ liver carcinoma cells had enhanced glycolysis (22). Osteosarcoma-initiating cells also showed a highly glycolytic phenotype (49). Breast CSCs demonstrated the upregulated glycolysis and simultaneously decreased OXPHOS (24). Heterogeneous results were showed for glioblastoma stem cells: Zhou et al. described highly glycolytic glioblastoma cells which were enriched for CSC populations by cell growth conditions (50) while Janiszewska et al. showed the importance of OXPHOS for CD133+ glioblastoma CSCs (28). OXPHOS, as the primary energy production pathway was also shown for leukemic (29), pancreatic (51) and ovarian (32) CSCs.

Many cancer cells demonstrate altered amino acid metabolism. For the majority of cancer cells, glutamine—usually a non-essential amino acid—becomes critically essential as they consume high amounts of it to cover their biosynthetic and energetic needs (52). The rewiring of glutamine metabolism in tumor cells is associated with specific genetic alterations including mitochondrial DNA (mtDNA) mutations (53), oncogenic KRAS (54, 55) and c-Myc overexpression (56). Glutamine enters cells *via* specific transporters (most of them belong to the alanine/serine/cysteine transporter (ASCT) family) and is used in various biochemical pathways. Bi-directional transporters of amino acids export glutamine in exchange for other amino acids (for example, cysteine). In the cytoplasm, glutamine is converted into glutamate and, subsequently, α-ketoglutarate (α-KG). Glutamate is a building block of glutathione—one of the main scavenges of reactive oxygen species (ROS), which protects the cells from oxidative injury and lethal DNA damage (57, 58). In glutamine metabolism, α-KG is an essential intermediate fueling tricarboxylic acid (TCA) cycle in mitochondria. Metabolites of the TCA cycle are, in turn, used for various other pathways, for example, nucleotide and fatty acid biosynthesis. Moreover, α-KG is a co-factor of the ten-eleven translocation (TET) family DNA demethylases and Jumonji-C (JMJ-C) family histone demethylases—enzymes that play a role in epigenetic regulation of gene transcription. Some pieces of evidence suggest that elevated α-KG to succinate ratio is a marker of stemness (59).

Another critical metabolic characteristic of cancer cells is their lipid metabolism. *De novo* lipid biosynthesis, enhanced lipid oxidation, and increased storage of lipids are unique characteristics of many cancers. For some of them, such as prostate cancer, lipid content was proposed as a potential biomarker, since the accumulation of lipids in prostate tissue of mice correlated with tumor stage (60). Increased lipid droplet content was shown for colorectal CSCs (61).

De novo lipid biosynthesis and fatty acid oxidation are among the most targetable features of CSCs (62, 63). CSCs from glioma (34) and pancreatic cancer (37) demonstrated upregulated lipogenesis; interesting that pancreatic CSCs fuelled their lipogenesis *via* enhanced glycolysis. Fatty acid synthase (FASN) is the critical enzyme in *de novo* lipid synthesis. Its expression is upregulated in many cancers, including lung, colon, breast, and ovarian cancer (64–67). SREBP-2, a transcription factor associated with *de novo* lipid synthesis, was shown to activate transcription of c-Myc in prostate cancer, therefore contributing to the increase of CSC properties (68). Increased fatty acid oxidation is critical for maintaining the stemness of breast cancer (69, 70) and leukemic cells (71).

## The Metabolic Interplay of CSCs and Tumor Microenvironment

**Figure 1:**
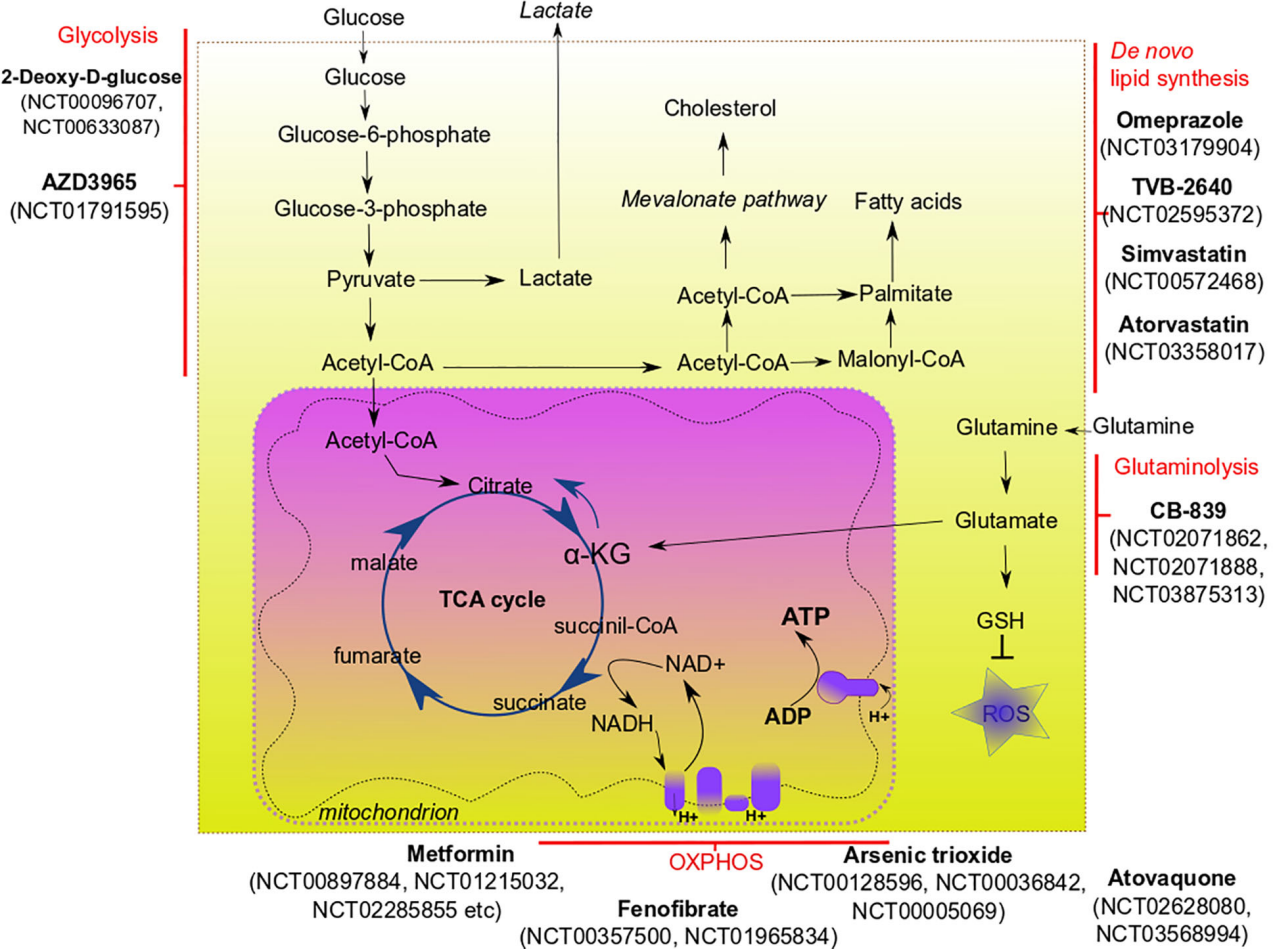
Main metabolic pathways of CSCs and their potential targeting in clinical trials.

Interaction of tumor microenvironment with cancer stem cells can support the survival and phenotype of CSCs. The tumor microenvironment consists of cancer-associated fibroblasts, endothelial cells, immune cells, extracellular matrix. Several factors are critically important for the sustaining of CSC metabolism, and hypoxia is one of them. Hypoxia is one of the major hallmarks of tumor microenvironment playing a critical role in CSC maintenance, quiescence, and therapy resistance (72). Hypoxia can affect CSCs in different ways, including activation of the hypoxia-inducible factor (HIF) mediated signaling that controls the tumorigenicity of CSCs (73). HIF-mediated signaling can interfere with the metabolism of cancer cells by upregulation of many glycolysis-associated genes, including glucose transporters from GLUT family (74). Pharmacological inhibition of GLUT-1 was shown to decrease the self-renewal properties of CSCs *in vitro* (75). Acidic microenvironment associated with hypoxic tumor areas is shown to promote CSC features by activation of the HIF-dependent transcription program (76). Interesting that cervical cancer cells located in hypoxic areas can produce lactate that is scavenged by cancer cells of oxygenated regions, fueling their proliferation (77). Cancer-associated fibroblasts (CAFs) can support the metabolic needs of cancer cells by feeding them via production of alanine (78), lactate, fatty acids or ketone bodies (79). CSCs from certain cancers (e.g., hepatocellular carcinoma and breast cancer) can promote angiogenesis and, therefore, increase nutrient supply, by releasing pro-angiogenic factors (such as VEGF) (80, 81). Tumor-associated immune cells contribute to the cancer progression and survival of CSCs *via* different mechanisms. Thus, cancer-associated macrophages can secrete various cytokines (e.g., TGFβ, IL-6) that induce the conversion of cancer cells to cells with CSC phenotype and contribute to chronic inflammation in tumor region (82, 83). Lactate produced by cancer cells in the hypoxic environment is known to induce conversion of tumor-associated macrophages into their pro-tumorigenic phenotype (84, 85). To survive under nutrient shortage conditions, CSCs may activate autophagy, the process of recycling their own nutrients by degrading organelles and large molecules. Enhanced autophagy as a pro-survival and pro-tumorigenic mechanism was demonstrated for breast (86), liver (87), osteosarcoma (88), and ovarian CSCs (89). Many of the above-described metabolic pathways confer CSC adaptation to the microenvironmental stresses, including a shortage of nutrients and anti-cancer therapies. These pathways are attractive targets for the eradication of CSC populations and better treatment outcomes (**Figure 1**, **Table 2**).

**Table 2:** Compounds for metabolic targeting of cancer stem cells.

Metabolic process	Compound	Cancer type	References
Glycolysis	Metformin	Hepatocellular carcinoma	(90)
		Prostate cancer	(91)
		Colon cancer	(92)
	2-deoxy-D-glucose	Triple-negative breast cancer	(93)
		Colon cancer	(92)
	Epigallocathechine gallate (EGCG)	Pancreatic cancer	(94)
Glutamine metabolism	CB-839	Triple-negative breast cancer	(95)
		Metastatic colorectal cancer	(96)
		Lung cancer	(97)
OXPHOS	Fenofibrate	Prostate cancer Liver cancer Glioma Breast cancer	Reviewed in (98)
	Arsenic trioxide	Acute promyelocytic leukemia	(99)
	Atovaquone	Hepatocellular carcinoma	(100)
		Breast cancer	(101)
	Rosiglitazone	Breast cancer	(102)
		Hepatocellular carcinoma	(103)
De novo lipid synthesis	Cerulenin	Glioblastoma	(34)
		Colon cancer	(104)
	C75	Breast cancer	(105)
	Omeprazole	Breast cancer	(106)
	Fatostatin	Prostate cancer	(107)
		Breast cancer	(108)

## Targeting CSC Metabolism

### Targeting Glycolysis

The most straightforward approach to inhibit glycolysis is to starve tumors for glucose. The effect on patients can be achieved by subjecting them to a ketogenic diet, containing low amounts of carbohydrates and balanced amounts of proteins and fat. Ketogenic diet-mimicking treatment *in vitro* effectively reduced CSC-signature in glioma cells (109). Experimental evidence showing the benefit of a ketogenic diet for cancer patients, especially those with glioblastoma and pancreatic cancer, prompted to investigate the potency of this approach as adjuvant therapy for these types of malignancy. However, current clinical data demonstrates mixed results (110). Although the ketogenic diet is usually well-tolerated, compliance with its strict regimes is generally challenging for patients; therefore, it is not considered as monotherapy, and even its usage as adjuvant therapy is discussable (111).

Compound-mediated targeting of glycolysis demonstrated better results in many preclinical studies. Metformin—an antidiabetic drug—has drawn recent attention in cancer research due to its ability to inhibit various molecular pathways leading to the elimination of cancer cells (112). Metformin attenuates glycolysis in a variety of tumor entities. Interesting that metformin can either downregulate glycolytic flux in hepatocellular carcinoma cells (90) or increase glycolysis in breast cancer cells (113). Moreover, it can also inhibit mitochondrial complex I, therefore impairing OXPHOS (114). Altering cancer cell respiration by metformin treatment led to a significant improvement in radiotherapy response in tumor xenograft models of prostate and colon cancer (91). Epigallocatechin gallate (EGCG) was tested as an inhibitor of glycolysis together with conventional chemotherapeutic drugs, and shown as a potent enhancer of chemotherapy (94). A synthetic analog of glucose, 2-deoxy-D-glucose, was tested *in vitro* and showed the ability to inhibit glycolysis and decrease the CSC phenotype of triple-negative breast cancer cells (93). Experiments on colon cancer cells demonstrated that a combination of 2-deoxyglucose with biguanides (such as 3-bromopyruvate) substantially reduced their proliferation (92). Deoxyglucose is now evaluated in clinical trials as a treatment agent for different cancers, such as lung, breast, and pancreatic cancer (clinicaltrials.gov numbers NCT00096707, NCT00633087).

### Targeting OXPHOS

OXPHOS is another promising metabolic target for CSCs. To date, many compounds have been designed to precisely target OXPHOS. Each compound targets a specific protein element of the electron transport chain blocking the transport of electrons and production of ATP. Most compounds that have shown their efficacy *in vitro*, *in vivo*, and in clinical trials, are directed towards mitochondrial complex I (115). The list of these compounds includes, but is not limited to metformin, phenofibrate, pyrvinium, rosiglitazone, pioglitazone, etc. Molecular mechanisms and efficacy of many OXPHOS-targeting compounds are described in reviews by Ashton and co-authors (115) and Sica et al. (116). Such OXPHOS-targeting compounds as atovaquone (clinicaltrials.gov No NCT02628080, NCT03568994), phenformin (NCT03026517) and arsenic trioxide (NCT00128596, NCT00036842, NCT00005069) are now under clinical trials for various solid tumors and leukemias. A combination of OXPHOS inhibition with other treatment modalities (particularly, radiotherapy) shows promising results *in vitro* and *in vivo* (117).

### Targeting Glutamine Metabolism

As an essential amino acid for most cancer cells, glutamine represents an attractive anticancer target: depriving cells for glutamine seems to be an effective therapeutic option. However, in reality, targeting glutamine metabolism is a challenging task. Systemic approaches to direct glutamine deprivation may be inefficient as glutamine can be synthesized *de novo* by non-cancerous tissues, such as muscles (118). Other amino acids, such as asparagine and arginine, may also contribute to cancer cell survival under gluatamine deprivation conditions (119, 120). Moreover, some components of tumor microenvironment (e.g. cancer-associated fibroblasts) are able to supplement cancer cells with *de novo* synthesized glutamine, supporting their proliferation (121).

Glutamine metabolism can be precisely targeted *via* blocking critical steps of glutamine utilization. One of the most potent targets is glutaminase 1 (GLS1)—the enzyme that converts glutamine to glutamate. Numerous *in vitro* studies showed that GLS1 was associated with cancer progression, metastasis and CSCs for hepatocellular carcinoma (41), triple-negative breast cancer (122) and pancreatic cancer (123). Inhibition of GLS1 disrupts redox balance in CSCs and can sensitize them to other types of therapy (e.g., radiotherapy) (97, 123). Several inhibitors of GLS1 have been developed, among them BPTES (124) and CB-839. After showing high efficacy *in vitro* (125) and *in vivo*, CB-839 entered clinical trials. Currently, CB-839 is tested in Phase I and II clinical trials alone or in combination with other chemotherapeutic drugs for such malignancies as leukemia, breast cancer, colorectal cancer, and lung cancer (NCT02071862, NCT02071888, NCT03875313).

### Targeting Fatty Acid Metabolism

As discussed above, the metabolism of fatty acids is substantially altered in many cancers. Cancer cells can be deprived of exogenous fatty acids or precursors for *de novo* fatty acid synthesis (such as glucose), which may be a promising strategy to slow tumor growth. Indeed, *de novo* fatty acid synthesis, which occurs in CSCs, but not healthy cells, seems to be one of the most promising targetable processes to eliminate the CSC population. Fatty acid synthase (FASN) is a target that received the most attention among all enzymes involved in the lipid metabolism of CSCs. Overexpression of FASN has been shown for a number of cancers, such as lung, prostate, ovarian and colon (66, 67, 126, 127). Inhibitors of FASN have pleiotropic effects on tumor cells, mostly because of the different pathways they can target. Cerulenin, a classical inhibitor of FASN, demonstrated high efficacy in reducing stem cell markers in glioblastoma and colon cells *in vitro* (34, 104). Chemical modifications of cerulenin, such as C75, were developed as the more stable analog of this drug, and C75 showed good results in inhibiting breast cancer cell proliferation (105). Such inhibitors of FASN as omeprazole and TVB-2640 are now evaluated in clinical trials for the treatment of breast cancer (NCT03179904, NCT02595372).

Not only FASN can be inhibited to target *de novo* lipid synthesis in cancer cells. Sterol regulatory element-binding proteins (SREBPs) are essential components of *de novo* lipid synthesis. A few compounds have been synthesized to target their functions. One of the most potent ones is fatostatin (128). It had a remarkable anti-tumor activity for prostate cancer; however, experiments with breast cancer cells showed mixed results, as fatostatin induced accumulation of both pro- and antiapoptotic lipids (108).

## Conclusions and Perspectives

Altered tumor metabolism is of utmost clinical importance as it mediates tumor resistance toward conventional anticancer agents, and metabolic co-targeting emerges as a novel, highly promising concept to enhance the efficacy of conventional treatment approaches. Metabolic inhibition of tumor growth by targeting CSCs is of specific interest as these cell populations are responsible for tumor maintenance and regrowth after treatment. Limitations of the current CSC assays and lack of the experimental models representing complex tumor microenvironments are a severe challenge to the development of the metabolic CSC-targeting approaches and their clinical translation. Many pitfalls also arise from the intratumoral heterogeneity of CSC metabolic features as well as the high plasticity of CSC nutritional demand during tumor progression and treatment. Future studies on heterogeneous CSC metabolic states at the level of single-cell resolution and employment advanced computational approaches to merge multi-omics data might yield clues for the development of novel metabolic targeting approaches and their implementation in current treatment regimens.

## Author Contributions

AM and AD contributed to the conception and design of the figure, tables, and manuscript. AM and AD wrote and edited the manuscript. All authors contributed to the article and approved the submitted version.

## Funding

Work in AD lab is partially supported by grants from Deutsche Forschungsgemeinschaft (DFG) (273676790, 401326337 SPP 2084: µBONE, and 416001651), from Wilhelm Sander-Stiftung (2017.106.1), BMBF (Grant-No. 03Z1NN11) and DLR Project Management Agency (01DK17047). This work was in part supported by Sächsischen Landesstipendiums for AM (Tyutyunnykova).

## Conflict of Interest

The authors declare that the research was conducted in the absence of any commercial or financial relationships that could be construed as a potential conflict of interest.
